# Effect of radiofrequency catheter ablation on left atrial structure and function in patients with different types of atrial fibrillation

**DOI:** 10.1038/s41598-022-13725-w

**Published:** 2022-06-09

**Authors:** Yue Liu, Qian Liu, Ying Yang, Chenfeng Zhang, Hongning Yin, Jinglan Wu, Lixia Yao, Lili Jin, Jing Yang, Liang Feng, Ruiqin Xie

**Affiliations:** 1 Division of Cardiology, The Second Hospital of Hebei Medical University, Shijiazhuang, Hebei People’s Republic of China; 2 Division of Cardiac Ultrasound, The Second Hospital of Hebei Medical University, Shijiazhuang, Hebei People’s Republic of China

**Keywords:** Cardiology, Interventional cardiology

## Abstract

Radiofrequency catheter ablation (RFCA) is widely used to treat atrial fibrillation (AF), but its effect on left atrial (LA) remodeling in patients with AF is not completely clarified. Few studies have reported the changes in structure and function of the left atrium in patients with different types of AF after RFCA. To analyze the effect of RFCA on the LA structure and function in patients with nonvalvular paroxysmal AF, persistent AF and long-standing persistent AF (LSPAF). RFCA was performed in 180 patients with paroxysmal AF, persistent AF and LSPAF. The changes of LA structure and function in echocardiogram and speckle-tracking echocardiography findings were compared before the procedure, and at 1, 2, 3, 4 weeks, and 2, 3, 6, and 9–12 months after the procedure. There were 60 patients in the paroxysmal AF group, 60 in the persistent AF group and 60 patients in LSPAF group. The pre-procedure LA diameter and volume were smaller in the paroxysmal AF group than persistent AF and LSPAF group. There was no significant change of in the LA structure and function in the paroxysmal AF group within 1 year. In the persistent AF and LSPAF groups, LA structure (anteroposterior diameter, LA volume) significantly decreased, but remained larger than that in paroxysmal AF group. In persistent and LSPAF, function (LA ejection fraction, strain, strain rate) increased significantly within 1 week, then gradually increased. RFCA improved the LA structure and function and resulted in heart reverse remodeling, especially for persistent AF and LSPAF.

## Introduction

Atrial fibrillation (AF) is the most common arrhythmia and is becoming an increasingly serious public health problem^1^. The main complications of AF are embolism and heart failure. AF is also an important risk factor for ischemic stroke and systemic embolism, and significantly increases the risk of stroke by five-fold^2^. Patients with AF who develop stroke have a poor prognosis, with high mortality and permanent disability rates. With the extension of AF duration, the left atrial (LA) diameter becomes larger and LA function declines, increasing the risk of heart failure^3^. As an effective treatment for AF, radiofrequency catheter ablation (RFCA) is widely used in clinical practice to maintain sinus rhythm, and improve cardiac function and quality of life, and is proven to be superior to anti-arrhythmic drugs^4,5^. However, RFCA still has a high-rate of relapse especially in patients with persistent AF and long standing persistent AF (LSPAF). Despite the widespread application of RFCA, its effect on LA remodeling in patients with AF has not been fully clearified. RFCA helps maintain sinus rhythm, which may improve LA function. However, RFCA can also cause iatrogenic myocardial damage and is even associated with new scarring resulting in LA dysfunction^6^. Previous studies performed in our center showed that there is no significant difference between cryoballoon ablation and RFCA in the effects on LA function in patients with paroxysmal AF^7^. However, few studies have evaluated LA remodeling in patients with different types of AF after RFCA. The overall LA function can be comprehensively evaluated by measuring the LA size with conventional echocardiography, and measuring the strain and strain rate (SR) with two-dimensional speckle tracking echocardiography (STE)^8^. The purpose of our study was to use echocardiography and STE to compare the changes in LA structure and function within 1-year after RFCA in patients with nonvalvular paroxysmal AF, persistent AF and LSPAF.

## Methods

This study was performed at the Second Hospital of Hebei Medical University and was designed to analyze the effect of RFCA on LA structure and function in patients with nonvalvular paroxysmal AF, persistent AF and LSPAF within 1-year. The protocol of this prospective cohort study was approved by the Ethics Committee of the Second Hospital of Hebei Medical University. All methods were performed in accordance with the relevant guidelines and regulations. All the patients met the diagnostic criteria of AF and written informed consent to undergo RFCA. A total of 434 patients who underwent RFCA in our department from April 2016 to August 2018 were enrolled. After the exclusion of 186 patients with incomplete data, 45 patients with recurrence and 23 patients with poor quality ultrasonic images, there were 60 patients in each of the three groups.

The study protocol was approved by the ethics committee of the Second Hospital of Hebei Medical University.

### Research subjects

According to the duration of AF, patients were divided into the paroxysmal AF group (n = 60), persistent AF group (n = 60) and LSPAF (n = 60). Paroxysmal AF was defined as relapse into sinus rhythm spontaneously within 7 days, generally lasting less than 48 h. Persistent AF was defined as AF that lasted for more than 7 days and required drugs or electric shocks to revert to sinus rhythm. LSPAF was defined as failure to revert to sinus rhythm for more than 1 year.

**Figure 1:**
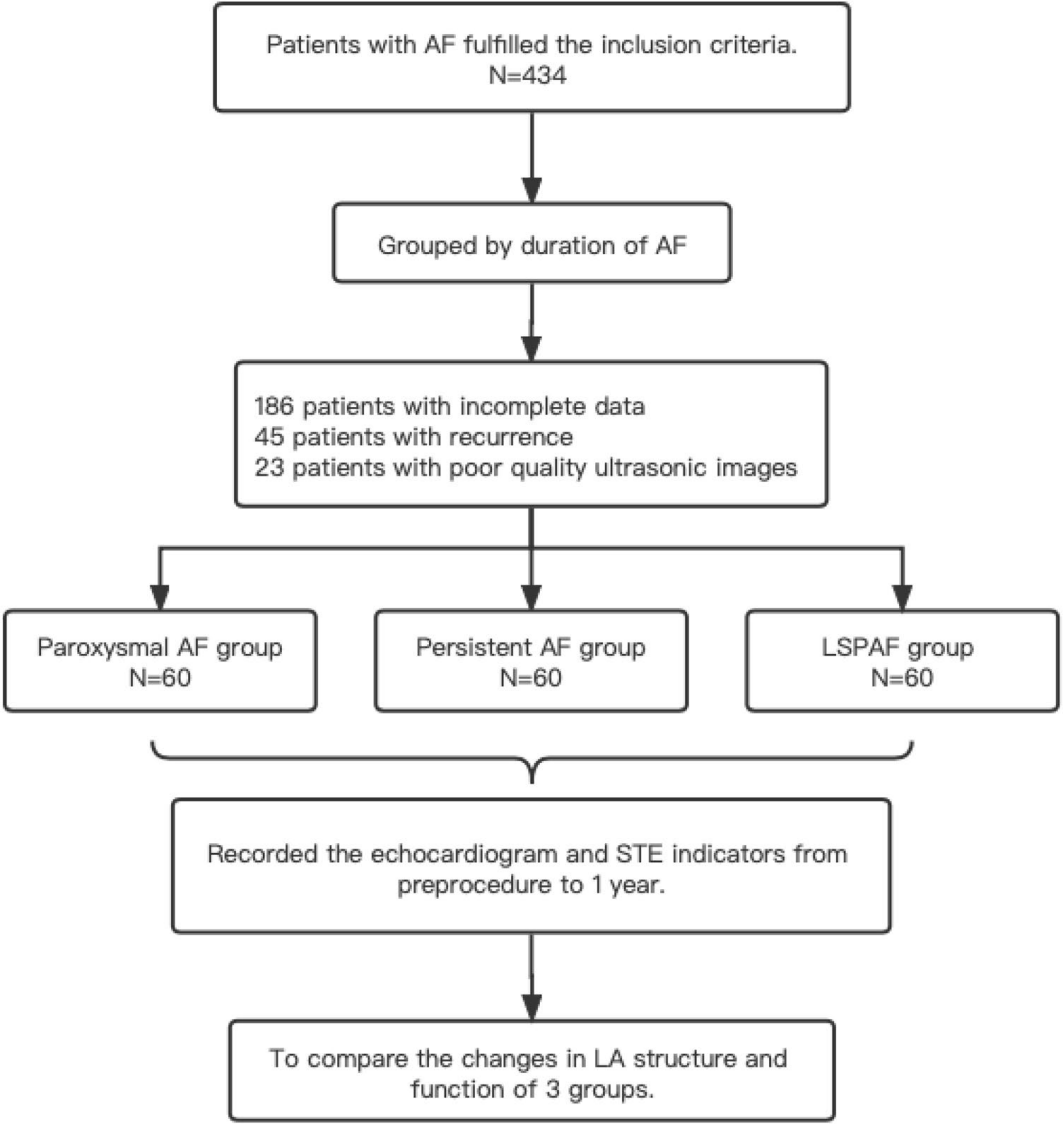
Study flowchart.

The flowchart of this study is shown in Fig. 1. The inclusion criteria were: (1) patients who met the diagnostic criteria of AF and consented to undergo RFCA; (2) age less than 80 years old. The exclusion criteria were: (1) evidence of an intracardiac thrombus on transesophageal echocardiography (TEE); (2) heart valvular disease (moderate and severe valve stenosis, severe valve regurgitation); (3) artificial heart valve replacement; (4) uncontrolled hyperthyroidism; (5) history of AF ablation; (6) pregnancy; (7) previous severe liver and kidney diseases, malignant tumors, and hematological diseases.

### RFCA procedure

Patients underwent cardiac computed tomography (CCTA) to evaluate the morphology and adjacent structures of the left atrial and pulmonary veins (PVs), and transesophageal echocardiography to exclude thrombosis of the LA and LAA before procedure. All procedures were performed under local anesthesia and conscious sedation. After septal puncture, the Carto 3 electrophysiological mapping system (Biosense Webster Inc., Diamond Bar, CA, USA) was used to assist in the reconstruction of the left atrial and PVs. The ablation catheter (ThermoCool SmartTouch; Biosense Webster) was inserted into the left atrium to perform RFCA. The mapping catheter (Lasso, NAV Ec, Biosense Webster) was used to record PV potentials. The procedural endpoint was defined as bilateral circumferential PV isolation following the standard procedure described in a previous study^9^.The absence of acute conduction recovery between the PVs and the LA was confirmed in each PV after waiting at least 20–30 min after the final ablation.

### Echocardiography and STE

Data were measured in all patients by a transthoracic echocardiography system (iE33 machines equipped with X3; Philips Medical Systems, Eindhoven, The Netherlands) and included the LA anteroposterior diameter, LA ejection fraction (LAEF), LA maximum volume (LAVmax) and LA minimum volume (LAVmin).Left atrial strain (S) and strain rate (SR) were measured by two-dimensional speckle tracking technique. We manually traced three markers in the LAVmax view to obtain SR curves and calculated the average value in the two-chamber view^10^. Three consecutive cardiac cycles with a stable echocardiographic appearance were recorded. The new parameters of S and strain rate SR in echocardiography can fully reflect the functional status of left atrial. S and SR are closely related to the amplitude and rate of myocrdial deformation. The strain rate is divided into three parts: left ventricular systole (SRs) reflects left atrial storage function, left ventricular early diastolic (SRe) responds to left atrial channel function, and left atrial systole (SRa) reflects left atrial pump function, the bigger absolute values, the left atrial myocardial elasticity better. The abovementiond data were measured before and after the RFCA procedure (at 1, 2, 3, 4 weeks, and 2, 3, 6, and 9–12 months). All ultrasound data were measured twice by professional sonographers, with the average value used in the analysis.

### Statistical analysis

Data were analyzed by SPSS Statistics, version 22.0 (Chicago, IL, USA). The K–S test was applied to determine whether the data were normally distributed. Continuous variable data followed normally distributed were presented as mean ± standard deviation. The Student's t-test was used to compare the mean valus of the three groups. The χ^2^ test was used for qualitative data. A p value < 0.05 was considered statistically significant. Continuous data of different indices before and after the RFCA procedure were assessed with analysis of variance for repeated measures, Bonferroni correction was performed on the statistical level α(α/8 = 0.05/8 = 0.006).

## Results

### Baseline data

All 180 patients underwent RFCA in our department from April 2016 to August 2018, including 60 patients in the paroxysmal AF group (mean age 56.9 ± 10.8 years, 34 men), 60 patients in the persistent AF group (mean age 61.3 ± 9.7 years, 37 men) and 60 patients in the LSPAF group (mean age 60.9 ± 7.7 years, 30 men). RFCA was successful in all patients. The basic characteristics of the three groups are compared in Table 1. There were no adverse events (included pericardial tamponade, stroke, bleeding, cardiovascular adverse and death) during follow-up.

**Table 1:** Baseline characteristics in 3 groups.

	Paroxysmal AF (n = 60)	Persistent AF (n = 60)	LSPAF (n = 60)
Age (years)	56.9 ± 10.8	61.3 ± 9.7	60.9 ± 7.7
Male, n (%)	35 (58.3)	37 (61.7)	30 (50)
Hypertension, n (%)	22 (36.7)	33 (55)	32 (53.3)
Diabetes, n (%)	5 (8.3)	5 (8.3)	14 (23.3)
MI, n (%)	3 (5)	3 (5)	2 (3.3)
HF, n (%)	6 (10)	29 (48.3)	23 (38.3)
Smoking History, n (%)	13 (21.7)	12 (20)	8 (13.3)
SBP (mm Hg)	134.5 ± 18	130.3 ± 13.8	128.1 ± 15.9
DBP (mm Hg)	81.7 ± 11.9	85.2 ± 11.6	87.8 ± 13.8
LVEF%	60.6 ± 4.7	59.7 ± 6.5	58.4 ± 7.6
CHA2DS2-VASc score	1.68 ± 1.32	2.48 ± 1.75	2.41 ± 1.73
Antiarrhythmic drugs (%)	41(68.3)	46(76.7)	49(81.7)

*AF* atrial fibrillation, *MI* myocardial infarction, *HF* heart failure, *SBP* systolic blood pressure, *DBP* diastolic blood pressure, *LVEF* left ventricular ejection fraction.

### Effects of RFCA on LA structure

**Figure 2:**
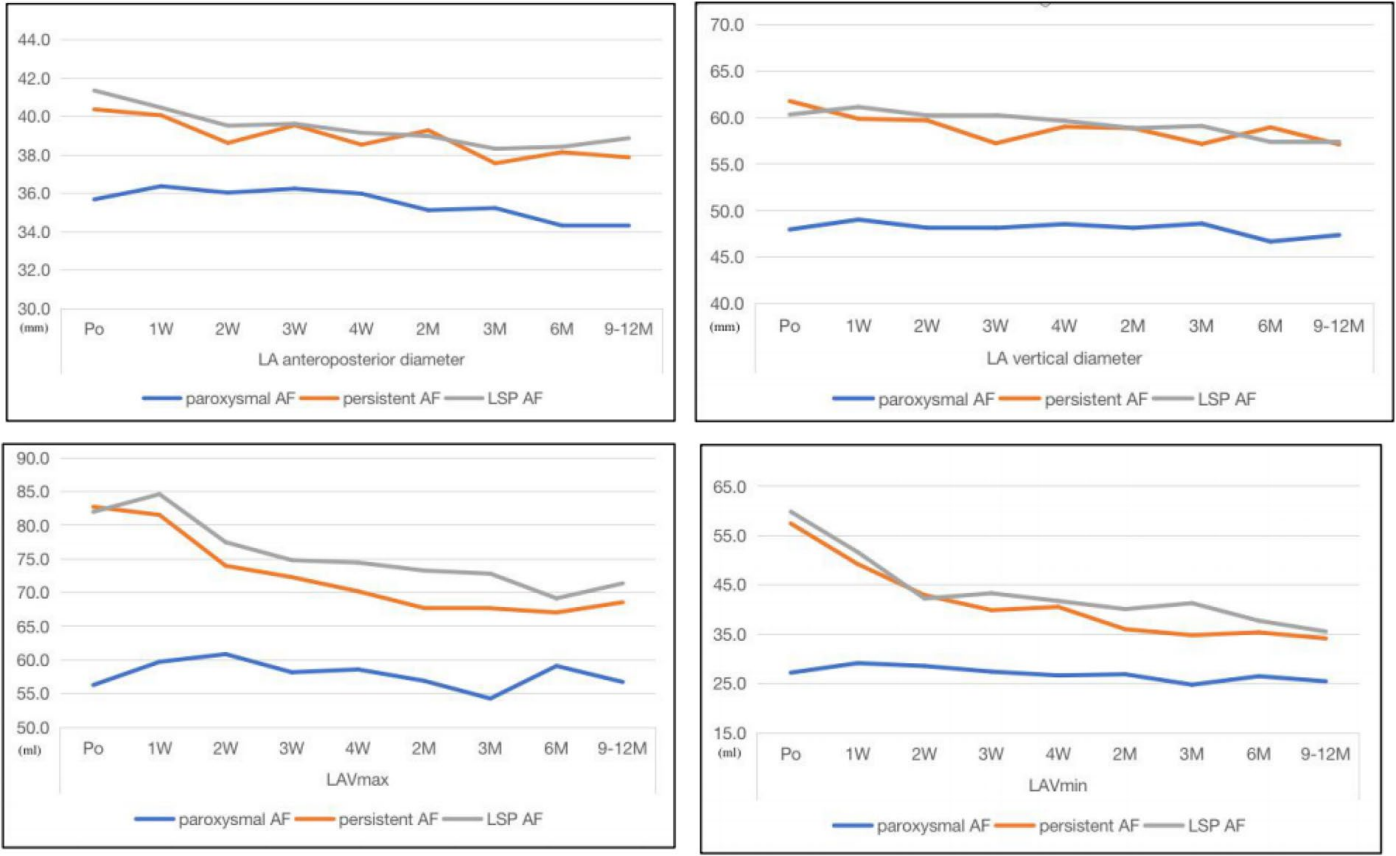
Effects of radiofrequency catheter ablation on the left atrial (LA) structure in patients with different types of atrial fibrillation (AF). *LSP* longstanding persistent, *LAVmax* maximum LA volume, *LAVmin* minimum LA volume.

The temporal changes in the LA anteroposterior diameter, vertical diameter and volume evaluated by conventional echocardiography in the three groups after RFCA are shown in Table 2 and Fig. 2.There was no significant change in the LA anteroposterior, vertical diameter and volume in the paroxysmal AF group within 1 year. In the persistent AF group, the LA anteroposterior significantly decreased from 3 months after RFCA (P < 0.006). In the LSPAF group, the LA anteroposterior diameter significantly decreased from 2 to 3 weeks, while there was no change in the LA vertical diameter. There was no significant change in the LA volume (LAV) in the paroxysmal AF group within 1 year. The LAV decreased most significantly in the persistent and LSPAF groups within 2 weeks after RFCA, and then decreased slightly. The pre-procedure LA diameter and volume were larger in persistent AF and LSPAF groups than the paroxysmal AF group. After RFCA, the LA diameter and volume decreased in the persistent and LSPAF groups, but remained larger than the paroxysmal AF group.

**Table 2:** Changes in left atrial (LA) structure after radiofrequency catheter ablation.

Variables	Groups	Po	1 W	2 W	3 W	4 W	2 M	3 M	6 M	9 M-1Y	P for group
LA anteroposterior diameter	Paroxysmal AF	35.68 ± 4.89	36.37 ± 4.73	36.03 ± 4.56	36.24 ± 5	35.98 ± 4.7	35.12 ± 4.22	35.23 ± 4.49	34.32 ± 5.59	34.3 ± 6.14	< 0.001
	p for time		0.19	0.51	0.30	0.57	0.30	0.40	0.01	0.01	
	Persistent AF	40.37 ± 4.15	40.06 ± 4.69	38.62 ± 4.71	39.54 ± 4.7	38.53 ± 5.03	39.28 ± 8.87	37.56 ± 4.94	38.14 ± 5.28	37.87 ± 5.77	
	p for time		0.65	0.01	0.23	0.01	0.11	< 0.001	< 0.001	< 0.001	
	LSPAF	41.35 ± 4.04	40.46 ± 4.27	39.52 ± 4.19	39.62 ± 3.79	39.15 ± 4.51	38.98 ± 4.06	38.32 ± 3.57	38.42 ± 3.68	38.87 ± 4.63	
	p for time		0.07	< 0.001	< 0.001	< 0.001	< 0.001	< 0.001	< 0.001	< 0.001	
LA vertical diameter	Paroxysmal AF	47.97 ± 9.7	49.03 ± 8.44	48.15 ± 7.94	48.13 ± 8.73	48.55 ± 9.04	48.14 ± 7.96	48.6 ± 9.23	46.67 ± 8.34	47.37 ± 8.32	< 0.001
	p for time		0.21	0.83	0.85	0.49	0.84	0.46	0.13	0.48	
	Persistent AF	61.78 ± 5.93	59.88 ± 5.38	59.71 ± 6.89	57.24 ± 6.68	59.02 ± 7.12	58.87 ± 7.26	57.17 ± 6.92	58.96 ± 8.72	57.12 ± 9.3	
	p for time		0.06	0.04	< 0.001	0.06	0.04	< 0.001	0.05	< 0.001	
	LSPAF	60.33 ± 7.34	61.15 ± 7.33	60.24 ± 6.63	60.27 ± 6.16	59.65 ± 6.35	58.87 ± 6.17	59.1 ± 6.1	57.39 ± 6.18	57.32 ± 6.93	
	p for time		0.37	0.92	0.94	0.45	0.11	0.17	0.01	0.01	
LAVmax	Paroxysmal AF	56.88 ± 16.54	58.08 ± 16.87	59.91 ± 17.35	58.09 ± 18.82	57.06 ± 19	56.07 ± 16.07	55.23 ± 16.32	56.03 ± 15.92	54.7 ± 13.72	< 0.001
	p for time		0.15	0.05	0.43	0.33	0.80	0.40	0.23	0.85	
	Persistent AF	80.86 ± 21.05	76.32 ± 21.04	72.6 ± 18.36	73.74 ± 20.99	69.81 ± 22.64	67.18 ± 18.31	69.73 ± 20.17	71.77 ± 21.42	71.18 ± 21.61	
	p for time		0.59	< 0.001	< 0.001	< 0.001	< 0.001	< 0.001	< 0.001	< 0.001	
	LSPAF	80.86 ± 17.43	78.55 ± 19.48	71.76 ± 16.12	71.49 ± 16.07	71.99 ± 20.16	71.36 ± 18.97	72.03 ± 19.11	68.59 ± 20.31	68.33 ± 18.25	
	p for time		0.25	0.05	0.002	0.001	< 0.001	< 0.001	< 0.001	< 0.001	
LAVmin	Paroxysmal AF	27.92 ± 11.6	28.75 ± 11.35	29.45 ± 11.38	28.5 ± 10.44	28.93 ± 9.29	27.87 ± 11.2	25.31 ± 10.37	25.71 ± 11.06	25.4 ± 9.6	< 0.001
	p for time		0.16	0.33	0.88	0.68	0.81	0.08	0.61	0.20	
	Persistent AF	60.71 ± 19.79	46.92 ± 15.05	44.68 ± 15.75	42.1 ± 13.49	39.1 ± 14.03	36.71 ± 12.58	35.18 ± 13.52	36.98 ± 14.97	36.48 ± 13.58	
	p for time		< 0.001	< 0.001	< 0.001	< 0.001	< 0.001	< 0.001	< 0.001	< 0.001	
	LSPAF	59.38 ± 13.37	48.29 ± 12.03	41.68 ± 11.21	38.42 ± 11.46	40.46 ± 13.99	37.87 ± 12.88	38.66 ± 12.87	39.49 ± 14.87	38.07 ± 12.96	
	p for time		< 0.001	< 0.001	< 0.001	< 0.001	< 0.001	< 0.001	< 0.001	< 0.001	

P values are given for the comparisons of variables at individual follow-up timepoints with their baseline values.

*LAVmax* maximum left atrial volume, *LAVmin* minimum left atrial volume.

### Effects of RFCA on LA function

**Figure 3:**
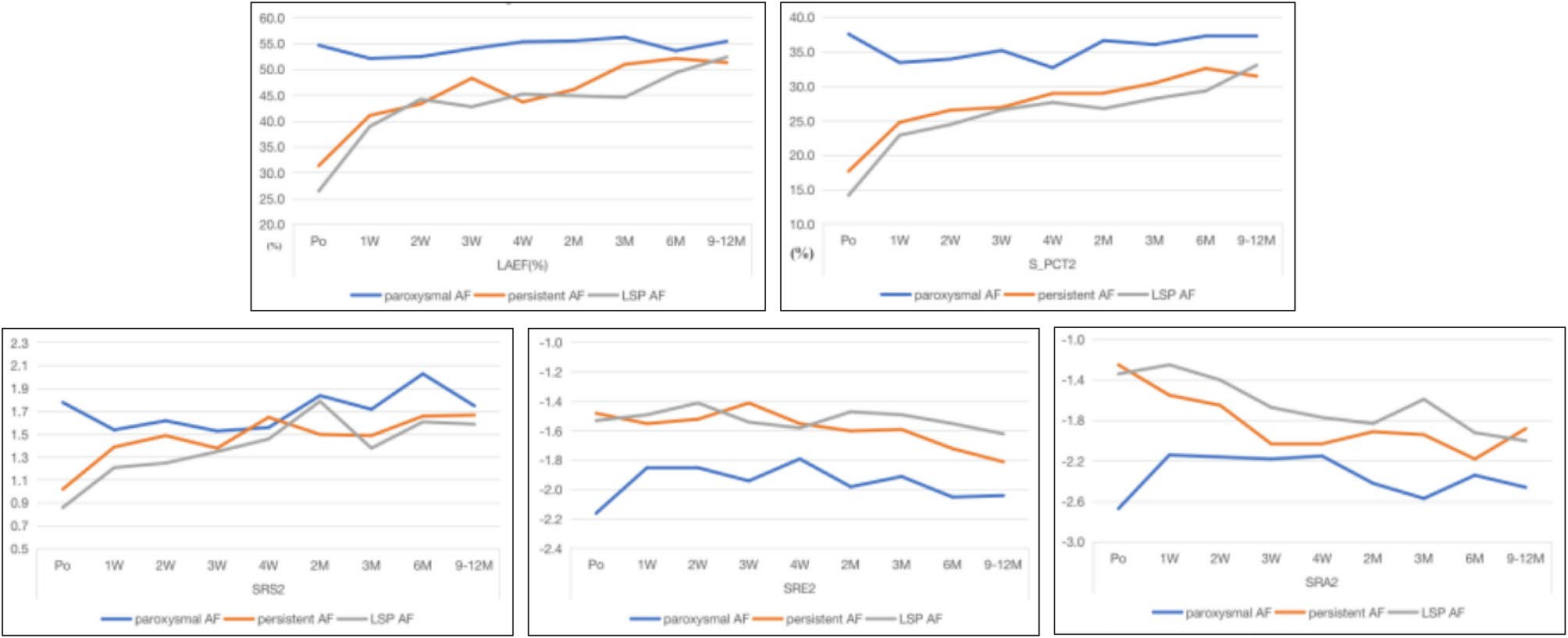
Effects of radiofrequency catheter ablation on left atrial function in patients with different types of atrial fibrillation (AF).

The LAEF, LA strain and SR at each follow-up timepoint in the three groups are shown in Table 3 and Fig. 3. There was no significant change in LAEF within 1 year in the paroxysmal AF group. The LAEF was significantly increased in the persistent AF and LSPAF 1 week after RFCA, and then increased sightly. In the paroxysmal AF group, there was no significant change in the LA strain in the two-chamber view within 1 year. In the persistent AF and LSPAF groups, the LA strain significantly increased after RFCA, especially within 1 week, and then increased slightly. The absolute values of the LA SR during left ventricular systole (SRs) and during LA systole (SRa) were significantly increased after RFCA compared with before the procedure in the persistent AF and LSPAF groups, the absolute values of the SR during early left ventricular diastole (SRe) did not significantly change compared with the pre-procedure value, but the absolute value had showed an upward increasing trend.

**Table 3:** Changes in left atrial function after radiofrequency catheter ablation.

Variables	Groups	Po	1 W	2 W	3 W	4 W	2 M	3 M	6 M	9 M-1Y	P for group
LAEF (%)	Paroxysmal AF	54.67 ± 15.34	52.13 ± 12.18	52.46 ± 11.95	54 ± 10.17	55.32 ± 10.27	55.51 ± 12.34	56.21 ± 11.58	53.6 ± 13.57	55.42 ± 10.87	< 0.001
	p for time		0.3164	0.3797	0.7789	0.7842	0.7418	0.5356	0.6857	0.7588	
	Persistent AF	31.39 ± 14.4	41.09 ± 14.19	43.34 ± 14.54	48.29 ± 15.11	43.71 ± 13.08	46.11 ± 13.56	50.97 ± 13.48	52.1 ± 14.16	51.32 ± 12.8	
	p for time		0.0003	< 0.0001	< 0.0001	< 0.0001	< 0.0001	< 0.0001	< 0.0001	< 0.0001	
	LSPAF	26.48 ± 10.2	38.96 ± 9.75	44.23 ± 11.28	42.78 ± 11.76	45.22 ± 12.6	44.92 ± 13.06	44.63 ± 12.94	49.36 ± 12.3	52.39 ± 12.41	
	p for time		< 0.0001	< 0.0001	< 0.0001	< 0.0001	< 0.0001	< 0.0001	< 0.0001	< 0.0001	
S	Paroxysmal AF	37.6 ± 13.19	33.46 ± 11.25	33.97 ± 10	35.22 ± 12.14	32.71 ± 10.34	36.66 ± 11.78	36.08 ± 10.62	37.33 ± 12.72	37.38 ± 11.53	< 0.001
	p for time		0.0668	0.0916	0.3062	0.0257	0.6796	0.4885	0.9091	0.9235	
	Persistent AF	17.67 ± 8.12	24.77 ± 9.96	26.55 ± 9.92	26.95 ± 10.41	28.98 ± 10.12	29.02 ± 10.78	30.49 ± 13.06	32.61 ± 11.91	31.49 ± 12.1	
	p for time		< 0.0001	< 0.0001	< 0.0001	< 0.0001	< 0.0001	< 0.0001	< 0.0001	< 0.0001	
	LSPAF	14.16 ± 5.42	22.9 ± 7.76	24.47 ± 8.01	26.59 ± 9.14	27.67 ± 8.5	26.78 ± 8.69	28.23 ± 9.79	29.34 ± 11.81	33.08 ± 12.25	
	p for time		< 0.0001	< 0.0001	< 0.0001	< 0.0001	< 0.0001	< 0.0001	< 0.0001	< 0.0001	
SRs	Paroxysmal AF	1.78 ± 0.63	1.54 ± 0.55	1.62 ± 0.48	1.53 ± 0.74	1.56 ± 0.48	1.84 ± 0.63	1.72 ± 0.54	2.03 ± 2.17	1.75 ± 0.44	< 0.001
	p for time		0.0296	0.1151	0.0451	0.0325	0.5857	0.5743	0.394	0.7692	
	Persistent AF	1.02 ± 0.38	1.39 ± 0.52	1.49 ± 0.47	1.38 ± 0.46	1.65 ± 0.75	1.5 ± 0.51	1.49 ± 0.82	1.66 ± 0.5	1.67 ± 0.53	
	p for time		< 0.0001	< 0.0001	< 0.0001	< 0.0001	< 0.0001	0.0001	< 0.0001	< 0.0001	
	LSPAF	0.86 ± 0.29	1.21 ± 0.41	1.25 ± 0.31	1.35 ± 0.43	1.46 ± 0.51	1.79 ± 2.57	1.38 ± 0.53	1.61 ± 0.63	1.59 ± 0.44	
	p for time		< 0.0001	< 0.0001	< 0.0001	< 0.0001	0.0069	< 0.0001	< 0.0001	< 0.0001	
SRe	Paroxysmal AF	− 2.16 ± 0.86	− 1.85 ± 0.75	− 1.85 ± 0.7	− 1.94 ± 0.93	− 1.79 ± 0.73	− 1.98 ± 0.89	− 1.91 ± 0.97	− 2.05 ± 1.02	− 2.04 ± 0.9	< 0.001
	p for time		0.0387	0.0316	0.1741	0.0119	0.2651	0.1334	0.5194	0.448	
	Persistent AF	− 1.48 ± 0.82	− 1.55 ± 0.76	− 1.52 ± 0.61	− 1.41 ± 0.59	− 1.55 ± 0.64	− 1.6 ± 0.62	− 1.59 ± 0.61	− 1.72 ± 0.59	− 1.81 ± 0.69	
	p for time		0.6144	0.7886	0.59	0.6028	0.3728	0.4164	0.0657	0.0201	
	LSPAF	− 1.53 ± 0.63	− 1.49 ± 0.5	− 1.41 ± 0.48	− 1.54 ± 0.47	− 1.58 ± 0.63	− 1.47 ± 0.52	− 1.49 ± 0.62	− 1.55 ± 0.63	− 1.62 ± 0.61	
	p for time		0.7555	0.2483	0.8962	0.6167	0.5808	0.742	0.8435	0.4064	
SRa	Paroxysmal AF	− 2.67 ± 1.13	− 2.14 ± 0.8	− 2.16 ± 0.79	− 2.18 ± 0.84	− 2.15 ± 0.83	− 2.42 ± 0.89	− 2.57 ± 0.97	− 2.34 ± 0.86	− 2.46 ± 0.68	< 0.001
	p for time		0.0036	0.0053	0.0083	0.0048	0.1724	0.5949	0.0756	0.2192	
	Persistent AF	− 1.25 ± 0.51	− 1.55 ± 0.68	− 1.65 ± 0.85	− 2.03 ± 0.92	− 2.03 ± 0.92	− 1.91 ± 0.74	− 1.94 ± 1.07	− 2.18 ± 0.92	− 1.88 ± 0.83	
	p for time		0.0066	0.002	< 0.0001	< 0.0001	< 0.0001	< 0.0001	< 0.0001	< 0.0001	
	LSPAF	− 1.34 ± 0.55	− 1.25 ± 0.41	− 1.4 ± 0.49	− 1.67 ± 0.59	− 1.77 ± 0.67	− 1.83 ± 0.66	− 1.59 ± 0.88	− 1.92 ± 0.81	− 2 ± 0.9	
	p for time		0.282	0.5172	0.002	0.0002	< 0.0001	0.066	< 0.0001	< 0.0001	

P values are for the comparisons of variables at individual follow-up timepoints with their baseline values.

*AF* atrial fibrillation, *LAEF* left atrial ejection fraction, *S* left atrial strain, *SRs* left atrial strain rate during left ventricular systole, *SRe* left atrial strain rate during left ventricular early diastole, *SRa* left atrial strain rate during left atrial systole.

## Discussion

Atrial remodeling affects LA structure and function and lays the foundation for the development of AF. Our study showed that the changes in LA structure and function after RFCA were different in three types of AF. Patients with persistent AF and LSPAF achieved significantly better improvements in LA structure and function than patients with paroxysmal AF, but still did not reach a similar level of LA structure and function to patients with paroxysmal AF at 1 year after RCFA. Our results suggest that patients with persistent AF and LSPAF are most likely to benefit from RFCA.

### Effect of RFCA on LA structure

Echocardiography can evaluate many structural indicators of the left atrium, such as LA diameter and volume, which are predictors of AF recurrence^11,12^. A previous meta-analysis showed that patients with a LA diameter of larger than 50 mm and a LAV of more than 150 ml are unlikely to maintain sinus rhythm after catheter ablation (CA) for AF^13^. Furthermore, LA diameter is reportedly an independent predictor of AF progression after RFCA^14^. Patients with a larger LA diameter (> 43 mm) are most likely to progress from paroxysmal AF to persistent AF, even after RFCA, suggesting that the large LA diameter results in severe electrical and anatomical remodeling, greater fibrosis and scarring, and higher LA pressure^14^. It is well known that LA dilation develops gradually from discoid to spherical^15^. LA spherical remodeling can be associated with increased atrial stretch. Chronic atrial stretch is a common etiologic factor in AF, and the electrophysiologic sequelae include slowing of conduction, prolongation of the effective refractory period, areas of low voltage and electrical scarring, double potentials and fractionated electrograms, and increased inducibility of AF.

Recent studies have found that the LAV is a more reliable predictor of AF recurrence than the LA diameter. A meta-analysis of 21 studies and a total of 3822 patients concluded that patients with AF relapse have a larger LAV and higher LAV index^16^. Each 1-ml increase in the LAV/LAV index is associated with a 3% increased risk of AF recurrence, and each 1.84-mm increase in the LA diameter is associated with a 0.8-ml increase in the LAV^16^. Kagawa et al.^17^ showed that the LAV decreases more significantly after catheter ablation in patients with persistent AF than in patients with paroxysmal AF (11.8 ± 12.8 cm^3^ vs. 4.0 ± 11.2 cm^3^; P = 0.04). Similarly, in our study, RFCA significantly reduced the LA diameter and LAV in patients with persistent AF and LSPAF, which may play a significant role in the maintenance of sinus rhythm after RFCA.

### Effect of RFCA on LA function

As the LA functional damage caused by AF precedes the change in LA structure^18,19^, the LAEF, S and SR may be more sensitive than the LA size in evaluating LA damage. LA enlargement leads to a decline in LA function, which represents maladaptive structural and functional remodeling and promotes electrical remodeling and an environment conducive to the occurrence of AF. Previous studies have demonstrated the importance of LAEF in the prognosis of cardiovascular disease. For example, LAEF is strongly associated with mortality and new-onset atrial arrhythmias^20^. A previous retrospective study also showed that a reduction in LAEF is an independent risk factor for AF recurrence after RFCA^21^. In the present study, the LAEF and LA strain did not change significantly in patients with paroxysmal AF within 1 year after RFCA, while the LAEF significantly increased in patients with persistent AF and LSPAF.

Normal LA function can be divided into three stages. Firstly, the left atrium serves as a reservoir to collect and store incoming blood during ventricular contraction. Secondly, the left atrium works as a conduit that empties passively into the left ventricle during early ventricular diastole. Thirdly, LA contraction works as a booster to promote left ventricular filling in the late ventricular diastolic stage, which enhances the intensity of ventricular contraction through the Frank-Starling mechanism^22,23^. These described LA functions can be quantified using STE assessments of strain and SR. The LA SR during left ventricular systole, left ventricular early diastole, and LA systole reflect the LA storage function, LA channel function, and LA pump function, respectively^24^. A decreased LA strain has been shown to correlate with LA wall fibrosis in AF patients. It has been reported that LA storage function is predictive of maintaining sinus rhythm in AF patients undergoing catheter ablation and is an an independent predictor of LA reverse remodeling^23^. The LA function was impaired to varying degrees in the patients with different types of AF in our study. In general, progressive LA fibrosis adversely affects the LA reservoir, conduit, and booster functions with the persistence of AF^22^. STE is an innovative imaging tool that can analyze and track myocardial segments, providing detailed information for the assessment of global and local cardiac function. Active deformation of the myocardium during the cardiac cycle can be assessed by STE strain imaging, a technique capable of identifying subtle cardiac dysfunction and heart muscle damage^11^. STE has recently been used to assess LA function and has been shown to be more useful than conventional echocardiographic variables as predictors of AF recurrence after RFCA^25^. There is reportedly a good correlation between the LA diastolic parameters measured by STE and the extent of LA fibrosis^26^, and STE may be helpful in the noninvasive assessment of LA fibrosis and selection of RFCA candidates.

Increased LA strain is an important factor in maintaining sinus rhythm after RFCA and is closely related to the left ventricular volume and diastolic function^27^. In patients with paroxysmal AF, there are improvements in LA remodeling and function, a decrease in the LA blood storage, and enhancements in LA booster and conduit functions at 3 months after RFCA^28^. An investigation into the role of LA strain in predicting recurrence of paroxysmal and persistent AF after RFCA found that impaired baseline global LA strain values measured by STE imaging appear to be associated with a higher rate of AF recurrence within 12 months after RFCA^25^. The present study also found that the baseline LA strain was lower in patients with persistent AF and LSPAF than in patients with paroxysmal AF, which may be associated with a heavier AF burden. However, the LA strain and SR of patients with persistent AF and LSPAF were significantly increased after RFCA, suggesting that RFCA has a positive effect on LA remodeling in these two patient groups. There was no significant change in the LA strain in the two-chamber view after RFCA in patients with paroxysmal AF, which further suggests that the positive effect of RFCA on LA remodeling is stronger in patients with persistent AF and LSPAF than in patients with paroxysmal AF.

## Conclusion

RFCA significantly improved the LA structure and function in patients with AF, resulting in reverse remodeling, especially in patients with persistent AF and LSPAF.

### Limitations

1. This was a single-center, prospective, cohort study with a small sample size. A larger sample size may better reflect the actual situation and make the curve smoother.
2. As the number of patients who complete follow-up is affected by many factors, the complete sample size for which data can be obtained is much smaller than the total number of patients undergoing surgery. The effect of RFCA on the LA structure and function requires confirmation in larger clinical studies.
3. More studies with longer follow-up are warranted to determine whether the patients obtain further benefits at more than 1 year after RFCA.

## Data Availability

The data that support the findings of this study are available from the corresponding author upon reasonable request.
